# Factors Affecting E-Shopping Behaviour: Application of Theory of Planned Behaviour

**DOI:** 10.1155/2021/1664377

**Published:** 2021-11-23

**Authors:** Honglei Tang, Zeeshan Rasool, Mohsin Ali Khan, Ahmad Imran Khan, Farooq Khan, Hina Ali, Anum Afzal Khan, Syed Arslan Abbas

**Affiliations:** ^1^School of Economics and Management, Huzhou University, Huzhou 310013, China; ^2^School of Economics and Management, Shaanxi University of Science and Technology, Xi'an 710021, China; ^3^National University of Modern Languages Multan Campus, 60000, Pakistan; ^4^Putra Business School, University of Putra, UPM, Serdang, 43400 Selangor, Malaysia; ^5^The Women University Multan, Multan, Pakistan; ^6^Faculty of Business, Law and Social Sciences, Birmingham City University, UK

## Abstract

E-shopping is a rapidly growing phenomenon among different individuals who intend to shop online. However, a trust deficit in the E-shopping environment has always been a critical issue in the brick-and-click mode of shopping, being one of the main reasons for E-cart abandonment in E-commerce. This empirical study is aimed at investigating the perceived effect of website trust on E-shopping intentions and behaviour, drawing upon the theory of planned behaviour (TPB). Data were collected through self-administered questionnaires from working adults who shop for garments online. Structural equation modelling was used to evaluate the model fit and assumptions. Our findings suggest that website trust and E-shopping attitude play substantial roles in building E-shopping intentions and actual behaviours. Both are the significant predictors of the behaviour mediated by E-shopping intentions. However, E-shopping intentions did not mediate between subjective norms and E-shopping behaviour, when working adults decide to purchase garments online.

## 1. Introduction

E-shopping is the process whereby customers directly buy goods or services from a seller in real time, without an intermediary service, over the Internet [1]. It is a form of E-commerce, which has become prosperous in communities where Internet-enabled devices have made online shopping easier for customers. The way that consumers purchase or check for appropriate items can easily shift. At present, customers are testing sites in a wide variety of ways, such as to gather information, compare product features and pricing to their near alternatives, and then pick the best available choices [2]. During the past two decades, the number of E-shopping retailers has increased significantly, indicating that, in the future, retailers will rely on this mode of shopping [3]. The manner in which retailers advertise and connect with their consumers has changed, as well as providing buyers and retailers with a global marketplace [4]. In Pakistan, the E-commerce boom has sparked the interest of Pakistani producers in concentrating on regions with high consumer opportunity [5].

Likewise, in South Asia, E-commerce has grown substantially in recent years; however, E-commerce in the region is still far below its potential [6]. In the Pakistani E-shopping context, most stores in Pakistan have developed websites where customers can shop online and can make payments through the use of debit/credit cards. Unfortunately, the general public in Pakistan has expressed a lack of confidence toward the goods that are presented to them online.

Pakistani adults, however, seem versatile and have increasingly engaged in online shopping, particularly for online food orders [7]. Although Pakistani communities have limited awareness of and trust deficit problems with E-shopping, individuals still find it to be a simple shopping source [8]. Digital tools provide consumers with easy access to information about the price, design, packaging, and characteristics of products. The ease of online shopping is an important aspect for people [9].

Despite its ease and convenience, a major problem associated with E-shopping is trust deficit, in which the buyers question the credibility of E-vendors and their E-shopping mediums [10]. Shopping cart abandonment due to trust deficit is a big challenge for E-vendors in the online shopping environment. While this phenomenon is rare in conventional brick-and-mortar offline environments, it is common in E-commerce. It has been statistically postulated that E-shoppers abandon their shopping carts nearly 69.6% of the time, costing E-vendors approximately US$ 61 billion in lost sales per annum [6]. “Trust deficit in E-shopping is one of the major reasons behind query abandonment” [11], resulting in a huge loss of revenues for E-vendors. Globally, garments are the most abandoned products (i.e., approximately 40%) among E-shoppers [12]. The cart abandonment rate for clothes/apparels has been found to be the highest among other commodities and has been gradually increasing since 2006. The Dawn online Pakistani newspaper report revealed that the risk of fraud and misleading practices has put a damper on E-commerce optimism [13]. “Customers fear that the actual product shown on the website might not be delivered” [14].

Hence, the current study intends to investigate the significance of website trust in the online shopping sphere, in order to assess how extensively trust can influence the behaviour of E-shoppers. E-commerce is a medium which allows consumers to purchase directly from producers or retailers by using an Internet web browser or social networking site (SNS). This direct contact between sellers and consumers has been enabled by the transition of the Internet to delivering information in global interconnection scenario. This work focuses on examining the confidence factor (as a perceived behavioural control) in E-shopping websites, along with two main histories of the expected theory of behaviour (i.e., behaviour and subjective norms). This factor (i.e., website trust) may enable or deter the performance of certain behaviours while using an E-shopping medium. The theoretical underpinnings of this study are based on the theory of planned behaviour (TPB). In psychology, TPB is a theory that links the beliefs and behaviour of an individual. The theory states that intentions toward attitude, subject norms, and perceived behavioural control together shape an individual's behavioural intentions and behaviours. TPB describes better that psychological activity is not necessarily voluntary and regulated by the individual. In the absence of confidence in E-shopping media, one does not have volitional influence over their actions, despite having two other primary determinants (attitudes and subjective norms). Trust is also the reciprocal confidence, through which the other party exploits the vulnerabilities of others in the course of an interaction [15]. A lack of confidence may lead to hesitation in E-commerce. In previous studies, trust has been established as a key element in effective online companies [16, 17].

There has been some research in Pakistan, given this changing trend, to investigate the factor of confidence in Internet technology as a platform for shopping [7]. This phenomenon will continue to rise in the future, due to the convenience factor of E-shopping. The significant roles of expectations and the expected actions in embracing E-shopping, therefore, need to be examined.

In the Pakistani context, garments have been observed to be one of the most preferred products to buy on the Internet, as compared to purchasing cell phones, laptops, or other electronic devices. Although the Pakistani community has limited knowledge about online shopping and also has trust deficit issues regarding the credibility of online shopping stores, people still consider it an easy source of shopping (Sulaiman et al., 2007). They may believe that the online shopping stores will not deliver the actual product that they advertise on their networking sites or even get the desired products tangibly in return for their online payments (Hassan et al., 2014). At the same time, a vast majority also consider that visiting an outlet physically is an exhaustive and cumbersome procedure, compared to online shopping, where they can get information regarding price, design, packing, and features through some digital interface while sitting in their bedroom (Phau et al., 2013). However, the transition from conventional to online shopping has been difficult in this region. The reason for this is not only the lack of trust of online shoppers in online shopping (e.g., that the vendor will not provide exactly what they advertise on their official site) and the expectation to be satisfied with their purchases (Hassan et al., 2014).

Our research questions are as follows:

*RQ1*. Does the E-shopping attitude of a working adult influence their E-shopping behaviour?

*RQ2*. Do the subjective norms of working adults determine their E-shopping behaviour?

*RQ3*. Does website trust affect the E-shopping behaviour of the working adults?

*RQ4*. Do E-shopping intentions mediate the relationship between E-shopping behaviour and its antecedents?

## 2. Literature Review

### 2.1. Subjective Norms

Subjective norms refer to the perceived social pressure to perform (or not to perform) a certain behaviour. The literature on subjective norms has indicated that the influence of subjective norms can provide equivocal results. Previous studies have concluded that someone who aims to follow people's expectations and wants to be the same would certainly have good subjective standards in E-shopping behaviour [18].

In the E-shopping literature, “however, there have been conflicting reports of subjective norms” [19]. Past studies have shown an important positive impact on consumer buying intentions by subjective norms [20, 21]. However, studies have also found a negative effect [22] or even no effect of subjective norms on the E-shopping intentions of customers. In the early stages of Internet adoption, a research by “trust and privacy” found that, in contrast to other technologies, such as telephone or email, arbitrary norms played no significant part [23].

The inconsistent findings within the subjective norm literature call for further research, in order to understand the generalizability of subjective norms in different contexts.

### 2.2. E-Shopping Attitude

Attitude is defined as a person's overall evaluation of a concept. Two types of attitude can be identified: attitudes toward objects and attitudes toward behaviours. As this study measures the attitudes of working adults toward E-shopping, attitudes toward behaviours are more relevant to the context of this study. An attitude toward behaviour refers to the “degree to which a person has a favourable or unfavourable evaluation or appraisal of the behaviour in question” [24], whereas a customer's attitude toward E-shopping refers to a “customers psychological state in terms of making purchases over the Internet” [25].

The psychological nature of customers in the context of an online shopping decision affects their attitude toward E-shopping. A research on the E-shopping behaviours of British and American consumers has also shown that E-shopping is a determinant of online shopping. Likewise, consumer research on E-shopping behaviour accepts that attitude represents a description of the positive or negative self-appraisal of a client's behaviour, values, feelings, and patterns during online transactions [26]. The better the behaviour of an individual is, in relation to the behaviour predicted, the more likely the person wants to participate in the behaviour.

### 2.3. Website Trust

Trust is a multidimensional concept which is complex in nature, and so, one may find a number of definitions of trust even in the literature relating to a similar context. Trust is the mutual assurance that, during an exchange, no party will exploit the vulnerabilities of another. Trust is the willingness of a person or group to be vulnerable to the actions of other group of people, based on expectations that the other will do a certain action benefitting the trust. Trust also refers to the belief of an individual in the trustworthiness of others, which can be determined by their perceived integrity, benevolence, and competence. Eventually, trust can be conceptualized as “the degree to which one can believe and rely upon promises made by others”. So, in context of online shopping, where the state of vulnerability of the user is quite high due to the dynamic disposition of cyberspace, trust has been theorized as a factor directly contributing to attitude [27]. Trust can be theorized as a belief that another individual or group will not behave opportunistically, for example, that a vendor will deliver exactly what has been promised [28]. Apart from various definitions, trust is usually considered essential in online shopping environments, as it consists of various kinds of potential risks which are associated to cyberspace.

As far as trust in the E-commerce domain is concerned, it leads to a belief that permits customers to voluntarily open themselves to actions of the E-sellers, after taking into account the E-seller's worth. This relates to the construct of trust as a belief encompassing goodwill and believability or honesty [29]. The E-commerce environment is uncertain, and thus, trust is more complex and important than in traditional commerce.

Trust is known as the confidence that an individual or group places in some entity; regardless of whether an individual's trust turns out to be well-employed or not, trust is instigated by the individual.

As recommended above, trust in an online business or transaction can occur as various trust (or trustee) relationships, but we confine our definition to be specific with one kind of trust association, that is, the trust that occurs for an individual toward a particular online shopping website. In this study, the object in our model is a website or an SNS that is browsed by consumers for transactional and/or informational purposes. Websites and social networking sites (SNSs) are referred to as the basic Internet technology that enables customers to interact with a website or the people behind the website. In the modern day, a website possesses both features, as it works as a storefront and also acts as a salesperson in the offline world. So, online trust was conceptualized as per the requirements of the current study, that is, “an attitude of confident expectation in an online situation of risk that one's vulnerabilities will not be exploited” [31].

The intention behind conducting this quantitative study was to evaluate all three determents of the behavioural theory (TPB) to know which antecedent is more influential to build behaviour while making purchase decision. Secondly, how can we increase the interest of the working adults to shop their garments by using some online shopping medium? This study intended to know the most influential factor among attitude, subjective norms, and website trust that leads to form intentions and then behaviours which eventually encourage or discourage the consumers to shop their garments online. This study helps to better answer the raised questions regarding consumers' behaviours that which factor highly motivates them to shop their garments online or otherwise?

In Pakistani context, garments are observed one of the preferred buying on Internet as compared to purchasing cell phones, laptops, or other electronic devices. Although Pakistani community has limited knowledge about online shopping and they also have a trust deficit issues on credibility of online shopping stores, people still consider it as an easy source of shopping (Sulaiman et al., 2007). They may think that online shopping stores do not deliver the actual product what they put in front of them on their networking sites or even they get the desired products tangibly in return of their online payments (Hassan et al., 2014). At the same time, a vast majority also consider to visit an outlet physically as an exhaustive and cumbersome procedure as compared to online shopping where they can get information regarding price, design, packing, and features through some digital interface while sitting in their bedrooms (Phau et al., 2013). For Pakistan, however, the transition from conventional to online shopping has been more difficult than the region. The reason is not only the lack of trust of online shoppers in online shopping that the vendor does not provide exactly what they put in front of them at their official sites and expect them to be satisfied with their purchases (Hassan et al., 2014). In current times, a phenomenal growth has observed in online shopping that surely indicates that in future, this mode of shopping will be the prime focus of the retailers. These indicators show that in near and far future, there is an enormous market potential of E-commerce growth is laying vacant for the current players and as well as for the new comers. Now the consumers are potentially more interested in adopting online shopping for their convenience which mold the producers and retailers to pay more focus on this area for growth and expansion of their businesses. The ease of online shopping forms as an emerging trend in Gen Y. The acceptance of online shopping has raised the retailers' interest for focusing on this area (Lim et al., 2015). According to Vijayasarathy and Jones (2001), by using Internet, buyer/produce interactive online shopping technology enabled buyers to have an opportunity to compare among desired products before making their purchase, whereas the second largest benefit of online shopping for consumers is to gain information about products and services besides accomplishing their purchases. E-commerce is a medium which allows the consumers to purchase directly from producers or retailers by using some Internet web browser or some social networking site (SNS). This sellers and consumers' direct contact did happen because of the transition of Internet for delivering information in global interconnection scenario.

Collectively, corporate reputation is conceptualized as the degree to which people or firms in the industry believe that a firm is honest and concerned about its customers. Therefore, perceived website reputation is characterized as “the degree of website popularity to which a consumer perceives”. However, website popularity and credibility are a mix of various other essential characteristics of an online website, such as legitimacy, uniqueness, visibility, transparency, and consistency. In online business, the good repute of a website plays a vital role in its significance and profitability. From the point of view of consumers, reputable and credible websites are more likely to be acknowledged among customers than unknown ones [32]. In fact, the websites which have significant reputation are probably more convincing than websites with low or no perceived trustworthiness. Therefore, consumer trust in a website is also affected by the opinions of their associates or referral cues, regarding the repute of the website.

Online shopping websites need to manage their image, as it is a valuable asset which usually yields high profitability. In context of online shopping, website image refers to the perception that the customers have in their mind regarding the website. It can also be defined as what customers perceive and what comes in their mind when they think about the website or see its logo [33]. Customer perception is the pivotal point that describes how a customer perceives the actions and procedures of an online shopping website. With regard to web-based shopping, perceived website image is also linked with some of the website physical and behavioural aspects, such as website visual appeal, layout, functionalities, the manners in which it collaborates with the customers, the variety of goods and service it offers, and, finally, its operational excellence for transactions.

### 2.4. E-Shopping Intentions

Intentions are presumed to be an indicator of the extent to which people are willing to approach a certain behaviour and how many attempts they will try, in order to perform that certain behaviour. A lack of intention to purchase goods online is the main obstacle in the development of electronic commerce [34]. Purchasing intention is a core aspect of consumer cognitive activity in the purchase of a particular product by a consumer [35]. Generally, when an individual has favourable attitudes or subjective standards or a highly perceived influence over their actions, their intention to enact an action will be stronger [36].

Although intention has been determined as a salient predictor of actual behaviour to shop online, it does not always translate into purchase action [37]. Based on TPB, perceived behavioural control determines the decision of an online shopper after online behavioural intention sinks in. A study on E-shopping intentions and behaviour found trust to be a major indicator in the replacement of perceived behavioural control, significantly influencing E-shopping intentions and behaviour [38]. Purchase intention may have a positive influence on actual online purchasing, and further investigation of the relationship between trust and intention in future studies has been recommended [39]. E-shopping behaviour is directly determined by E-shopping intentions, which are influenced by the E-shopping environment.

As per recommendations and a step ahead from the base study where the sample was centric to undergraduate and postgraduate students of one of the renowned postgraduate institutions in Perlis, Malaysia, this study has taken the “working adults” as a study subject for originating the behavioural intent of the working class that contrary to the students has better power to purchase and sovereign in making their decisions and they do not depend upon other family earning heads. Additionally, website trust has taken as an additional construct in online shopping scenario for measuring its impact as a perceived behavioural control. Hence, the above criterion is to get ensured by using some screening questions that the respondents have easy access over digital media. They are employed somewhere and free to make their purchase decisions and thus have experienced of buying their garments in past through some social networking site (SNS) or online shopping store. Intention refers to the willingness of an individual to perform certain behaviours (Chen, SheenLou, 2006). It also refers to the strength of intention that how strong it is, in performing a certain behaviour (Venkatesh et al., 2003). Researchers, for instance Hennington et al. (2009), have paid attention to behavioural intention. TAM is one of the better accepted models for understanding desire and willingness to use a technology (Schepers & Wetzels, 2007). As per Yu et al. (2005), perceived ease of use and perceived usefulness both define the individual's attitude significantly, which mean customer's feelings toward using online shopping.

Venkatesh et al. (2003) have found that the attitude has no direct effect on intention. Meanwhile, according to the most renowned theories like TRA (Fishbein & Ajzen, 1975), TAM (Davis et al., 1989), and TPB (Ajzen, 1991), attitude has more significant positive impact on Intention, whereas many other researchers who conducted their researches on similar framework underscored the attitude's strong impact on behavioural intent (e.g., Cheong & Park, 2005; George Joey, 2002; Jiang, Chen, & Wang, 2008; Kumar & Ghodeswar, 2015). Nakagawa and Gouvêa (2010) and Gouvea (2010) suggested that attitude is a determinant related to intention to adopt E-shopping. Venkatesh et al. (2003) have revealed in his study that how significantly the intentions determine usage behaviour. In such context, outcome of the aforesaid statements may summarize as, attitude has a significant positive influence on intentions. In other words, consumer's favourable and positive emotions toward online shopping resulted in increases of consumers' willingness for online shopping.

### 2.5. Theoretical Model

**Figure 1:**
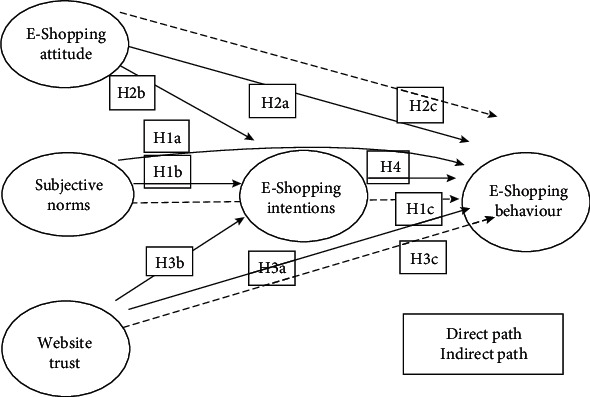
Diagrammatical representation of relationship between study variables (theoretical model).

For better understanding of the research hypothesis, Figure 1 presents the theoretical model, aimed at investigating the relationships among the study constructs in the case of online garment shopping.

As per the theoretical framework of the research (Figure 1), hypotheses were developed to investigate the research objectives. Hypotheses are used to designate the logical relationship that is imagined between two or more variables in a formal statement, which can be tested through some statistical operation. Icek Ajzen's theory of planned behaviour states that subjective norms signify the perceived social influence for performing (or not performing) a behaviour. It is the impact of an individual's normative beliefs that motivate them to approve (or not) a specific behaviour. More precisely, it refers to an individual's perception about whether society think they should involve in given behaviour or not. Therefore, we developed the following hypotheses:

Hypothesis 1a (H1a). Subjective norms affect the garment E-shopping behaviour of consumers.

Hypothesis 1b (H1b). Subjective norms affect the garment E-shopping intentions of consumers.

Hypothesis 1c (H1c). Subjective norms affect the garment E-shopping behaviour of consumer, through the mediating role of E-shopping intentions.

Chih-Chung and Chang [40] analysed six past studies that measured the attitude toward online shopping and confirmed that all studies showed a significant positive influence of online shopping attitude on online purchase intention and behaviour. According to TRA (Fishbein & Ajzen, 1975), TAM (Davis et al., 1989), and TPB (Ajzen, 1991), attitude has a significantly positive effect on behavioural intent. Numerous researchers have confirmed this relationship (e.g., Bruner & Kumar, 2003; Chang & Wang, 2008; Chen, Sheen, & Lou, 2005; Chen, Sheen & Lou, 2006; Cheong & Park, 2005; George, 2002; Lin, 2007; Shin, 2007). Hence, it was hypothesised that

Hypothesis 2a (H2a). Attitude affects garment E-shopping behaviour.

Hypothesis 2b (H2b). Attitude affects garment E-shopping intentions.

Hypothesis 2c (H2c). Attitude affects garment E-shopping behaviour, through the mediating role of E-shopping intentions.

Chen and Tan (2004), Yu et al. (2005), Wu and Chen [30], Cho and Fioritto (2009), and Lee and Park [32] have established that trust has a significantly positive influence on behaviour. So, it was hypothesised that

Hypothesis 3a (H3a). Website trust affects garment E-shopping behaviour.

Hypothesis 3b (H3b). Website trust affects garment E-shopping intentions.

Hypothesis 3c (H3c). Website trust affects garment E-shopping behaviour, through the mediating role of E-shopping intention.

Lim et al. [3] also revealed that purchase intention exerts a significantly positive impact on online shopping behaviour. Thus, we hypothesised the following:

Hypothesis 4 (H4). E-shopping intention influences garment e-shopping behaviour.

## 3. Research Methodology

### 3.1. Research Design

In the survey questionnaire, data comprising two parts were obtained. The first segment consisted of surveys and information about the Internet use, familiarity, and experience of the respondents. The second component consisted of five-point Likert measurements, which varied from extremely disagree to strongly agree.

A data screening procedure was applied to the data collected through the self-administrated questionnaire. First, missing data, outliers, normality, and interitem correlations were computed for each item. Second, the adequacy of covariances and Cronbach's alpha were computed for each construct. Lastly, a confirmatory factor analysis was conducted for each of the constructs.

After the data screening process, the conceptual model was translated into an AMOS model, consisting of a measurement part (confirmatory factor analysis, CFA) and a structural equation part (structural equational modelling, SEM). In order to evaluate the complex structural relations, the model consisted of complex structural relations between variables and so, a structural equational modelling (SEM) technique was used. SEM allows for the construction of a coherent dependent link chain between a number of structures to be observed simultaneously, while taking measurement errors into account [41]. Consequently, the methodology indicates the manner in which the observed variables refer to the latent constructs and their unified dependency.

### 3.2. Research Measures

We adopted measurement scales from the literature. These multi-item instruments have already been tested for measuring the same concepts and were found to be valid and reliable for use in a similar study setting.

Subjective norms refer to the “perceived social pressure to perform or not to perform the behaviour in question.” The first four items (Quests. # 1−4) of the questionnaire were measuring subjective norms, as adopted from [42]. These items were measured using a five-point Likert scale, with answers from “strongly disagree” to “strongly agree.” The subjective norm scale indicated a fair level of reliability, with Cronbach's alpha (*α*) of 0.747, at the level also endorsed by Robert A. Peterson (1994) in his study “A Meta-Analysis of Cronbach's Coefficient alpha,” in which he took various constructs from numerous past studies that discussed the personality and behavioural traits of individuals. He found, in his meta-analysis, that the average mean of Cronbach's alphas varies across studies, depending upon the nature and the environment of the study. The average mean of the Cronbach's alpha (*α*) for subjective norms, after considering 289 studies, was 0.76.

Attitude toward behaviour is operationally defined as the “degree to which individuals have favourable or unfavourable appraisal of the behaviour of interest.” Questions no. five to eight measured the impact of online shopping attitude on purchase intentions and behaviour. These four items (Quests. # 5−8) were adopted from Taylor and Todd (1995). A five-point Likert scale, with answers from “strongly disagree” to “strongly agree,” was used for measuring these four questions. Online shopping attitude scale indicated a good level of reliability, with Cronbach's alpha (*α*) of 0.804 (Table 1).

Trust implies the “degree to which one can believe and rely upon promises made by others.” Trust in the E-commerce sphere relates to the belief that encourages customers to voluntarily open themselves to actions through the E-seller, after considering the seller's worth. This relates to the formation of trust as a belief encompassing goodwill and believability. To measure the trust of respondents in websites, we adopted 5 items (Quests. # 9−13) from [43]. These five items were also measured on a five-point Likert scale, with answers from “strongly disagree” to “strongly agree.” The website trust scale had a good level of reliability, with a Cronbach's alpha (*α*) of 0.847 (Table 1).

Purchase intentions refer to the “willingness of a person to buy the required products or services.” In the E-commerce context, online purchase intention can be theorized as “a situation when an individual need to purchase the required products or services through the website.” Four items (Quests. # 14−17) were employed for measuring the online purchase intentions of the respondents, as adopted from [44], which were again measured using a five-point Likert scale. The online purchase intention scale possessed a good level of reliability, with a Cronbach's alpha (*α*) of 0.855 (Table 1).

Consumer shopping behaviour can be conceptualized as “the sum of a consumer's attitudes, preferences, intentions and decisions regarding the consumer's behaviour in the marketplace when purchasing a product or service.” For measuring online shopping behaviour, 17 items (Quest. # 18−34) were used, adopted from [45]. All items were gauged using a five-point Likert scale with answers from “strongly disagree” to “strongly agree.” The online shopping behaviour scale also showed a good level of reliability, with a Cronbach's alpha (*α*) of 0.847 (Table 1).

### 3.3. Procedure/Data Collection

The current study was designed considering population statistics with a considerable sample size (*N* = 500) from 2019 to 2020, in order to gain statistically valid results. Using closed-ended questionnaires, E-shoppers who had used the Internet as a shopping tool in previous times went through the quantitative study process. Working adults who were 20 years or older and who had shopped for garments online were taken as the unit of analysis. A purposive (nonprobabilistic) sampling technique was employed for extracting the sample, as the target population had particular traits (i.e., age, working status, and past E-shopping experience). The data presented in this paper were collected from five hundred respondents through self-administered questionnaires. A total of 470 questionnaires were returned producing a 94.5% response rate, and 439 were considered adequate for data analysis after data screening—33 questionnaires had some missing values and so were excluded from the final sample. Overall, 93.4% of questionnaires were adequate for further analysis.

### 3.4. Participants

A population is an overall collection of individuals or a subject, which is the prime focus of the scientific query, which sample is extracted to measure the variables of interest. The population normally refers to a particular community that observes common binding characteristics or traits.

As per the predefined objectives of the study, the target population of concern was working adults in Pakistan, who were purposively selected as the subject of analysis. The target population of the study included both males and females who purchased their garments online, through online store/website or social networking site (SNS).

A total of 439 E-shoppers responded to the survey, consisting of 360 male and 79 female working adults. The group composition of the participants included different rates of sex, age, marital status, and education. All respondents were asked to choose whether or not to comply with the statements of questions from a list of responses.

## 4. Data Analysis and Discussion

### 4.1. Sample Demographics/Respondent Profile

In Table 2, the mean values shown indicate the central tendency of the data, where the acquired values were clearly spread around a central value which perfectly described the data. The low standard deviation values indicate that the data points tended to be close to the mean of the data set, showing relatively close agreement among respondents and little variation about the answers of the questions. The mean and standard deviation values verified the significance of the data, and that there were very small chances of error.

The mean values shown in Table 3 demonstrate the central tendency of the data, where the acquired values were clearly spread around the central values. The standard deviation is a measurement of how widely the responses are spaced. From the above table, it can be that standard deviation was very small, indicating that the data points tended to be close to the mean of the data set. Hence, it can be assumed that there was relatively close agreement among the respondents mentioned in Table 4. All answers were relatively close to the mean, with just a little variation, proving that the data were very significant and there was a very low chance of error. Therefore, the responses acquired using the items of the online shopping behaviour subscale direction significantly varied from each other.

### 4.2. Multivariate Normality

Skewness and kurtosis tests verified the multivariate normality and ensured that the results of the study could not be skewed by significant variations in the main data. The results showed that the data were uniformly distributed, as neither the skewing values (peakness) nor kurtosis (flatness) surpassed their normal range [46].

As we dealt with a sample for further analysis in this study (i.e., it is a population in terms of the EFA in Table 5), the Principal Axis Factoring method with direct oblique rotation was carried out using the 34 items. In Exploratory Factor Analysis (EFA), the factors are permitted to be correlated with one another in promax rotation. The factor pattern matrix contained the coefficients for the linear combination of the variables.

The abovementioned Table 6 presents the factor pattern matrix, which contains the coefficients for the linear combination of the variables.

### 4.3. Validation of the Measurement Model

A measurement model was used to link the observed variables with the latent constructs, while the instrument scores and the concepts that they are meant to measure were linked through confirmatory factor analysis (CFA). Before undertaking the confirmatory factor analysis, the convergent and discriminant validities of study instruments were assessed, to determine how thoroughly these constructs gauged the intended concepts.

The convergent validity obtained from the six factors with low factor loads in the measurement model and other loadings in the factor exceeding the threshold value (0.70) was demonstrated using the criterion provided by [47]. Construct reliabilities exceeding 0.70 were considered and, after removing the poor factor loadings, the average variance extracted (AVE) was upgraded to an acceptable level (i.e., ≥0.50, ranging 0.51–0.62), as shown in Table 1. So, all factors satisfied the discriminant validity and were precise in nature (i.e., truly measuring the characteristics being represented by the variables).

To ensure the uniformity and stability of the measures, the internal and the composite reliabilities were measured. The Cronbach's alpha test confirmed the internal consistency and reliability of the concepts, with values ranging from 0.75 to 0.86 (Wollack, Cohen, & Wells, 2003). The Composite reliability values, ranging from 0.80 to 0.93, were also above the proposed level (0.70) [48]. Through the empirical data shown below, the convergent validity was also verified.

The correlation matrix was constructed, in order to observe the interconstruct correlations. It indicated that these variables were not mutually correlated with each other. The bivariate test variables were below the suggested threshold value (<0.7) [49]. Both attitude toward shopping and confidence on the web appeared to be highly positive (*r* = 0.67 and *r* = 0.63, respectively), which provided reasonable relationships with E-shopping activity (*r* = 0.54 and *r* = 0.49, respectively). Contrary to the other constructs, subjective norms had weak correlations with E-shopping intentions and E-shopping behaviour (*r* = 0.19 and *r* = 0.23, respectively) mentioned in Table 7.

Multicollinearity was measured by examining the tolerance and Variance Inflation Factor (VIF). The VIF statistics in Table 8 show the predictor variables were moderately correlated. All research constructs had VIF values less than the threshold value (<3) and higher than 1.

Kaiser–Meyer–Olkin (KMO) and Bartlett's tests mentioned in Table 9 confirmed the adequacy and suitability of the data. Taken together, these tests satisfied the minimum standard which should be passed before conducting CFA on data. The KMO values of all five study constructs were greater than the recommended range (>0.6) and closer to 1, showing the adequacy of percentage of variance in data. Thus, we confirmed that the sampling was adequate, and the data for all study constructs was suitable for conducting confirmatory factor analysis (CFA).

### 4.4. Model Measurement

A five-step process, consisting of model definition, description, estimation, evaluation, and amendment, was carried out for the study model. First of all, the latent variables with their indicators were listed, and error terms were also described in the model specification. The model established that it had enough information on the equation to generate unknown parameter estimates. The estimation of various model fit indices, such as GFI, AGFI, CFI, TLI, and RMSEA, was used to estimate the hypothesised model parameters. Chi-square (*χ*^2^) and some other signs were added, in order to assess the model's degree of accuracy, as the chi-square value (*χ*^2^) is sensitive to a large sample (*n* > 200). As a rule of thumb, some other indices, such as GFI, CFI, NFI, and RMSEA, may clarify the fit pattern if the value of chi-square/Df. is less than 3. Finally, the model was respecified by codefault terms, and some restrictions in path coefficients were enforced. The importance of fit indices, which otherwise showed a bad fit, was achieved, and the fit was improved. Therefore, other assumptions were made, namely, that there were no parity restrictions on the factor loadings for these measures.

The chi-square statistic (*χ*^2^) was below the minimum value (i.e., CMIN/Df.<3), which verified the latent construct's distributions to be substantially different. The values of GFI, AGFI, CFI, and TLI, which showed the overall fitness of this model, were higher than 0.9. The RMSEA analysis of a population involves the root mean square error approximation; when the RMSEA value is below 0.07, a model can be considered appropriate.

The fit indices of the model were respecified by showing that the model hypothesised had a good fit to the data (*N* = 439, *p* < 0.001, GFI = 0.908, AGFI = 0.924, CFI = 0.910, TLI = 0.929, and RMSEA = 0.060). With the overall fit statistics, due to important and practical indications, the hypothesised model was presumed to be very strong for the current data. All elements that contribute significantly to their constructs were assisted by the predicted relationships between the constructs and their indicators.

Table 10 shows overall model fit summaries for the original and revised models. Some assumptions were taken into account for these indicators; for example, no equality constraints were set on the factor loadings. As per the overall measurement results of the actual model of the study (where *N* = 439, *p* < 0.001, GFI = 0.864, AGFI = 0.838, CFI = 0.899, TLI = 0.886, and RMSEA = 0.055) demonstrated an average fit of the model overall. So, this average fit led to the need for model respecification.

### 4.5. Structural Equation Modelling (SEM)

#### 4.5.1. Structural Model Assessment

For testing the study hypotheses, a bootstra*p* value of 2000 resamples was calculated using standardised route coefficients. To obtain the same number of estimates, due to longer alignment, a large number of replicates were needed. The mean was less than the target value in the current analysis, so the test statistics may have also fell into one critically significant area. Thus, due to the expectation of both forms of interactions (i.e., positive or negative), two-tailed values and 95% confidence intervals were taken into account. The findings of the data analysis indicated that the path structure for the study variables (direct and indirect) was accurate and adequate. The findings of the hypothesis tests are summarised in Table 11, where the path coefficients and *p* values of the study variables describe the direct, indirect, and complete influences.

The structural model analysis found that, aside from arbitrary criteria, there were two other structures—E-shopping attitudes and confidence in the website—that had significant explicitly positive effects on E-shopping behaviour. Eight out of ten findings of the analysis were supported by the final statistical tests. Interest indicates that the E-shopping satisfaction is a key factor in actual online purchasing actions. E-shopping intentions often effectively clarify and mediate the relation between the independent variables (i.e., attitude to E-shopping and confidence on the website) and the dependent variable of the analysis.

#### 4.5.2. Hypothesis Discussion

Hypothesis H1a indicated the positive relation of subjective criteria to E-commercial behaviour. The SEM findings showed good support for the importance (*β* = 0.091, *p* < 0.05) of hypothesis (H1a) and indicated that subjective regulations have a significant positive connection to E-shopping behaviour. Hypothesis H1b concluded that this relationship did not support (*β* = 0.012, *p* < 0.05) the connection between social norms and E-shopping intentions early in the analysis. Therefore, it was not endorsed, as no significant correlation between subjective norms and E-shopping intentions existed. H1c, therefore, did not endorse the findings, as no relevant indirect relationship existed (*β* = 0.003, *p* < 0.05) between E-shopping and subjective expectations through the mediator.

We verified successful direct (H2a) and indirect (H2c) E-shopping–attitude relationships, wherein positive relationships were formed (*β* = 0.233, *p* < 0.01 and *β* = 0.128, *p* < 0.01, respectively). Hypothesis H2b suggested the relationship between attitude and E-shopping intentions. Our findings supported this substantially positive relationship (*β* = 0.452, *p* < 0.01) and demonstrated that E-shopping is a vital predictor of online buying intentions.

Finally, the hypotheses H3a and H3c postulated that there exist direct and indirect relationships between website trust and E-shopping actions. Such favourable relationships (*β* = 0.154, *p* < 0.01 and *β* = 0.098, *p* < 0.01, respectively) were verified by our findings, in that trust in a website is a successful predictor of E-shopping activity. A correlation between confidence and a mediator (E-shopping intentions) was suggested by hypothesis H3b. This substantially favourable relationship was confirmed by our findings (*β* = 0.347, *p* < 0.01).

## 5. Conclusions and Findings

Our findings demonstrated the strong influence of E-shopping intentions on actions and indicated that E-shopping intentions effectively clarify and serve as mediators between E-shopping conduct and its context. Therefore, those aimed at developing E-shopping actions of working adults should, in particular, focus on E-shopping intentions. These results are compatible with those of previous similar studies (Hsu & Bayarsaikhan, 2012; Lim et al., 2015; Orapin, 2009; Pavlou & Fygense, 2006; Roca, Garcia, & Jose, 2009). However, E-shopping intention did not act as a mediator between subjective standards and E-shopping conduct, as no significant direct relationship between subjective standards and E-shopping intentions was observed, at least, for the working sphere of E-shoppers.

Therefore, as was originally assumed in hypothesis H1c, no partially mediating or indirect connection between subjective norms and E-shopping behaviour was observed. All proposed hypotheses except for H1b and H1c were endorsed, as no significant connection with the DV through mediating between subjective standards and E-shopping intentions was created.

The statistical analysis of the data showed that social expectations, E-shopping location, and trust in websites are all significant factors that influence the E-shopping behaviour of consumers, which ultimately leads to an Online Shopping Purchase. Therefore, the situation is very different from that of other parts of society, in the event of apparel E-shopping plans for working adults. Subjective expectations did not create substantial positive or negative relationships with intentions, unlike E-shopping attitude and website confidence. Therefore, along with subjective criteria, these predictors contribute to compliance. The findings of the analysis, therefore, did not support the hypotheses H1b and H1c. Many previous studies (see, e.g., Chua et al., 2006; Jamil & Mat, 2011; Tseng et al., 2011) have predicted these relationships to be lacking. More specifically, the inconsistent relationship between subjective standards and expectations is the most important and frequently discussed weak point linked to the TPB. The founder of the theory (Ajzen, 1991) also explained this deficiency, by suggesting that motives are primarily influenced by behaviours and behavioural regulation of an individual's traits. The results of this study are, therefore, also related to previous research, in that the subjective expectations did not influence the actions of adults working for the purchase of their online equipment. While other social groups, such as students or housewives, may be effectively assisted or affected by their significant peers when deciding whether to participate in or not participate in such behaviour, it has been indicated that, once the customer has agreed to shop online, no more input is considered through other paths (e.g., from their social circle or peer group).

There were important direct and indirect relations between E-shopping actions and the mediator and, subsequently, the dependent variable. In terms of E-shopping mindset, its effects on mediators (E-shopping intentions) were positive both directly and indirectly (H2a and H2c), being substantially positive for E-shopping conduct. The relationship between E-shopping and E-shopping expectations showed a good relationship. The results demonstrate that E-shopping reflects the E-shopping activity of working adults, in order to pursue E-shopping as a way to purchase their clothes. E-shopping mindset is a key determinant of the goals and actions of E-shopping. The interviewees generally had a favourable evaluation and usually promoted their conduct.

The findings of the study were finally confirmed through hypotheses H3a, H3b, and H3c, all of which were significantly positive, both explicitly and indirectly, focused on DV (E-shopping behaviour) and explicitly for to the intermediary (E-shopping intentions). These results indicated that confidence in a website is an expanded construction which is ideally relevant to recognise, in the sense of E-shopping. The fact that consumers can shop or give up their shopping cart is an important factor. E-shopping consumers become more relaxed as their confidence in E-shopping media (e.g., a website) increases.

Trust plays a key role in defining the conduct of E-shopping, as it transforms the good expectations and actions of consumers, in order to create E-shopping requests for online shopping. Some previous studies have confirmed confidence to be a basic demand for growth in E-commerce (Mukherjee & Nath, 2007; Sutanonpaiboon & Hamimah, 2010); in particular, in 2012 (Hsu and Bayarsaikhan, 2012; Jiang, Chen & Wang, 2008). Around the same time, the literature noted that a confidence deficit is the key reason why E-shopping is not chosen as a shopping medium or why requests are abandoned. The dynamic disposal of cyberspace is very high, due to the insecurity of users (Whyte, 2016).

Eventually, when a customer visits a shopping website to check for the correct items, the E-shopping cycle begins. This quest either transforms into a real buying transaction or not, which is a secondary problem. He et al. (2008) concluded that the biggest obstacle for the growth of E-commerce is the absence of online purchasing intentions. Generally, several attempts are made to perform a certain activity, which is probably the main reason for the leaving of carts. Expectations are, therefore, possibly the best indicators to show how ready consumers are to purchase online. As Dolatabadi et al. [42] reported, the E-shopping intentions of consumers have major effects on their own purchasing decisions. Three key antecedents—social standards, attitude, and perceived power and expectations—influence the actions of TPB.

### 5.1. Contributions of the Study

Our theoretical contribution expands the literature by assessing the effects of website trust on the intentions and behaviours of consumers related to E-shopping, which has never been tested before in such a context and setting. In replacing PBC with website trust, we have extended the literature and, hence, proved the significant determinant of choosing E-shopping in the TPB setting. We have shown that “website trust” is worth consideration as a contributing factor that builds favourable intentions and behaviours toward E-shopping, rather than the opinions of significant peers (in the case of working adults).

In practical terms, this research provides valuable insight into the E-shopping preferences of E-shoppers (adult workers), for the advancement of relevant marketing strategies. We recommend that E-vendors design viable systems which support and attract customers toward E-shopping, through persuading them to believe that the E-vendor is honest and concerned about their customers. This is essential, as most customers question the integrity and trustworthiness of the E-vendor while performing an E-transaction. Thus, E-vendors must convince users to have trust in their shopping websites, as it is obvious that trust significantly influences the intent and behaviour related to E-shopping. Our findings suggest that E-shopping websites need to develop more trust in transactions for their clientele, in order to attract and motivate them more to build positive E-shopping behaviours.

This paper is intended as a guide for the transformation of E-commerce for companies attempting to project their businesses online. Such businesses should focus even more on the prestige of their E-shopping newspapers, which they would otherwise have overlooked.

### 5.2. Limitations of Study

There were several limitations to this study. The research sample was restricted to an explicit section of the population (working adults) and a particular geographical region; therefore, it may not have attracted students, young people, or housewives, and it may have produced some specific results, such that expanding these findings to other segments in the business is suggested. Second, we focused primarily on the E-shopping conduct related to the apparel industry. Ultimately, it is not necessary to draw or extend these findings to certain segments of consumer markets, such as electronics, beverages, cosmetics, books, or foodstuffs. Finally, in the constructs used to illustrate the decision to shop online, there were no additional variables, such as the fear of potential scarcity or the peculiar emotional state of individuals, such as enjoyment, disgust, or disdain with respect to their actions of interest. Such variables may lead to certain behaviours or not to conduct the interesting behaviour.

In relation to other online consumer goods, the proposed conceptual model should also be tested. Culture affects behaviours, thus driving demographic change interdependently and complementally (Pollak & Watkins 1993). For that reason, the potential E-commerce trends that have a cross-cultural influence or a particular demographic trend should be considered, which may be focused on specific sex or low-income groups, as well as students. The next assessed demographic may be housewives and students, as they tend to rely on their family heads. Likewise, it may be beneficial to increase the sample size and to adjust the geographical location.

### 5.3. Research Implications

The results of this study offer a better understanding of E-trust and recommend E-vendors to design a viable online selling system that supports and encourages positive and secure feelings toward E-shopping. Firstly, E-vendors should focus on strengthening these feelings by increasing the trustworthiness of their E-shopping medium (e.g., website). Secondly, the E-vendors must assure their customers that they will not behave opportunistically and will deliver the promised products and services to them. Such commitments and promises will reduce the uncertainty and add additional value in E-shopping. However, these results may not generalize to other geographical areas or social classes as a whole. Finally, this study opens up some new frontiers in support of future research relating to behavioural intent in the online shopping context.

In comparison to the former studies, this study has portrayed an improved explanatory power of two of the main components of behavioural sciences like intention and online shopping behaviour in a specific context. This study theoretical contributes further illustrative strength in explaining the reasons of variation in consumers' argumentative purchase intentions. The study also expands the theory by applying the effects of website trust on consumers' intentions and behaviours to shop their products and services through some online shopping medium. This study can also assist managers in recognizing and eliminating the potential key behavioural obstacles and allows them to deliver highly customer oriented online customized services and as well as to enlarge their loyal customer base by increasing trustworthiness of their shopping websites. Further, it also delivers guidance for future research, for focusing on the strengths and eliminating the weaknesses. Similar to others, this study also has some weak points which need to cater through further examination in this sphere. So, the results may not generalize to other geographical areas or social classes as a whole. Eventually, this study opened up some new frontiers in support of future research for knowing behavioural intent in online shopping context.

## Figures and Tables

**Table 1:** Measurement model result summary.

Component	Items	Main loading	AVE	Composite reliability	Cronbach's alpha
E-shopping attitude	ATT2		0.580	0.802	0.804
		0.616			
	ATT3	0.828			
	ATT4	0.821			
			0.577		
Subjective norms	SN1	0.676		0.803	0.747
	SN2	0.805			
	SN3	0.791			
Website trust			0.507	0.837	0.847
	WT1	0.683			
	WT2	0.691			
	WT3	0.709			
	WT4	0.715			
	WT5	0.761			
E-shopping intentions	ES11	0.839	0.629	0.871	0.855
	ES12	0.728			
	ES13	0.776			
	ES14	0.825			
E-shopping behaviour	ESB1	0.675	0.510	0.931	0.847
	ESB2	0.794			
	ESB3	0.676			

Note. AVE: average variance extracted.

**Table 2:** Measurement of study variables.

Variables name	Measurement scale names
Subjective norms	Domain specific innovativeness (DSI), attitude, subjective norms, planned behaviour (Dolataba et al., 2012)
E-shopping attitude	Perceived usefulness, ease of use, attitude, and behavioural intension (Taylor and Todd, 1995)
Website trust	Loyalty intensions, perceived trust, overall satisfaction, attributive service satisfaction, perceived trust (Chiou, 2004)
E-shopping intensions	Online purchase intensions, consumer attitude, information availability, online shopping motivations (Vazquez and Xu, 2009)
E-shopping behaviour	Accessibility of data, customer perception, service and infrastructural, nondelivery risk, financial risk, perceived behaviour control, return policy (Javaide et al., 2012)

**Table 3:** Demographic distribution of sample (*N* = 439).

Sample	Sample	No. of valid	
Characteristics	Classifications	Cases (*N*)	Percentage (%)
Gender	Male	360	82.0
	Female	79	18.0
Age	20−26 years old	159	36.2
	27−33 years old	153	34.9
	34−40 years old	99	22.6
	41−45 years old	28	6.40
Marital status	Single	189	43.1
	Married	246	56.0
	Divorced	4	0.90
Education level	Secondary school	46	10.5
	Bachelor's degree	146	33.3
	Master's degree	185	42.1
	Above master's degree	38	8.70
	Other (diploma, etc.)	24	5.50

Note. Descriptive statistics (*N* = 439), their classifications, and frequencies.

**Table 4:** Descriptive statistics of the study.

Variables Names	*N* statistics	Minimum statistics	Maximum statistics	Mean statistics	Std. deviation statistics	Variance statistics
SUBNORM	439	1.00	5.00	3.6703	0.6536	0.427
ESATT	439	1.50	5.00	3.6959	0.7493	0.561
WEBTR	439	1.00	5.00	3.5164	0.8019	0.643
ESINT	439	1.00	5.00	3.5729	0.8083	0.653
ESBHVR	439	1.76	4.94	3.5247	0.5264	0.277
Valid *N* (list wise)	439					

**Table 5:** Frequencies.

		OSATT	SUBNORM	WEBTR	OPINT	OSBHVR
*N*	439	439	439	439	439	439
	0	0	0	0	0	0
Skewness		−0.710	-1.034	−0.527	−0.771	−0.614
Std. error of skewness		0.117	0.117	0.117	0.117	0.117
Kurtosis		0.189	1.023	−0.452	0.348	0.602
Std. error of kurtosis		0.233	0.233	0.233	0.233	0.233
*p* value		0.001	0.022	0.01	0.001	

Note. Skewness value > +1 or <−1: balanced distribution and kurtosis value < 1: flat distribution.

**Table 6:** Factor pattern matrix.

	Factor pattern matrix					
	1	2	3	4	5	6
SN3L	0.067	0.022	0.062	0.066	−0.036	0.475
SN4L	0.081	−0.104	0.090	0.009	−0.030	0.742
ATT1L	0.052	−0.121	0.031	0.084	0.664	−0.052
ATT2L	0.085	−0.017	0.050	0.010	0.672	0.001
ATT3L	0.093	0.537	0.025	−0.075	0.252	0.074
ATT4L	−0.174	0.476	0.122	−0.002	0.431	0.024
WT1L	−0.118	0.074	0.664	−0.085	0.203	0.019
WT2L	−0.103	0.097	0.649	−0.094	0.120	0.128
WT3L	0.115	−0.143	0.728	0.059	0.034	−0.010
WT4L	0.105	0.209	0.491	0.034	−0.020	−0.076
WT5L	−0.014	0.139	0.728	0.044	−0.159	0.074
ESI1L	0.050	0.731	−0.079	0.034	0.151	−0.042
EI2L	−0.097	0.766	0.014	0.114	−0.110	0.072
ESI3L	0.138	0.812	0.081	−0.100	−0.128	−0.138
ESI4L	0.064	0.753	0.125	0.010	−0.167	−0.038
ESB1L	−0.028	0.222	−0.052	0.566	0.072	0.011
ESB2L	−0.051	0.119	−0.012	0.604	0.032	0.056
ESB3L	0.219	−0.076	−0.021	0.439	0.160	0.030
ESB4L	−0.038	−0.075	−0.002	0.928	−0.014	−0.027
ESB5L	−0.026	−0.048	0.021	0.824	−0.013	0.024
ESB6L	0.676	−0.122	0.202	0.100	−0.032	−0.065
ESB7L	0.531	−0.011	0.157	0.099	−0.017	−0.120
ESB8L	0.799	−0.098	0.039	−0.128	0.105	−0.033
ESB9L	0.723	0.005	−0.004	−0.105	0.061	0.054
ESB10L	0.515	0.080	−0.133	0.014	−0.001	0.133
ESB11L	0.672	0.171	−0.326	0.009	−0.003	0.100
ESB13L	0.362	0.096	−0.015	0.108	0.232	−0.079
ESB14L	0.504	0.065	0.096	−0.044	−0.097	0.132
ESB15L	0.319	0.207	0.178	0.155	−0.092	−0.012

**Table 7:** Pearson correlations matrix.

MODEL	SN	ESA	WT	ESI	ESB
Subjective norms	1				
E-shopping attitude	0.239^∗∗^	1			
Website trust	0.216^∗∗^	0.622^∗∗^	1		
E-shopping intentions	0.194^∗∗^	0.670^∗∗^	0.630^∗∗^	1	
E-shopping behaviour	0.235^∗∗^	0.540^∗∗^	0.497^∗∗^	0.554^∗∗^	1

Note. ^∗∗^Correlation is significant at the 0.01 level (2-tailed).

**Table 8:** Multicollinearity statistics.

		Collinearity statistics	
	Model	Tolerance	VIF
1	(Constant)		
	E-shopping attitude	0.479	2.089
	Subjective norms	0.935	1.069
	Website trust	0.527	1.896
	E-shopping intentions	0.476	2.100

Dependent variable: E-shopping intentions.

**Table 9:** KMO and Bartlett's tests.

KMO and Bartlett's	E-shopping attitude	Subjective norms	Website trust	E-shopping intentions	E-shopping behaviour	Overall
Kaiser–Meyer–Olkin (measure of sampling adequacy)	0.740	0.747	0.827	0.779	0.856	0.904
Barlett's test of sphericity (Approx. chi-square)	595.9	648.1	873.2	781.9	2863.9	6941.2
Df.	6	6	10	6	136	561
Sig.	0.000	0.000	0.000	0.000	0.000	0.000

Note. Value (KMO of >0.5 or ideally >0.7) for adequacy of percentage of variance [50].

**Table 10:** Model fit summary (original and revised model indices).

Model	Items	CMIN/Df.	Df.	GFI	AGFI	CFI	TLI	RMSEA
Original model	34	2.333	500	0.894	0.868	0.899	0.916	0.055
Revised model	28	2.580	328	0.908	0.924	0.910	0.929	0.060

Note. GFI: Goodness of Fit Index; AGFI: Adjusted Goodness of Fit Index; CFI: Comparative Fit Index; TLI: Tucker–Lewis Index; RMSEA: The Root Mean Square Error of Approximation.

**Table 11:** Hypothesis testing result summary.

Hypothesis	Relationships	Path coefficients	*p* value	CI	Results
H1a	SN 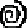 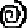 ESB	0.091^∗^	0.022	0.013–0.170	Supported
H1b	SN 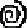 ESI	0.012^∗^	0.766	−0.064–0.092	Not supported
H1c	SN 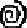 ESI 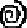 ESB	0.003^∗^	0.761	−0.017–0.027	Not supported
H2a	ESA 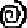 ESB	0.233^∗∗^	0.001	0.106–0.357	Supported
H2b	ESA 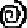 ESI	0.452^∗∗^	0.001	0.360–0.539	Supported
H2c	ESA 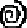 ESI 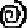 ESB	0.128^∗∗^	0.001	0.067–0.196	Supported
H3a	WT 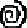 ESB	0.154^∗∗^	0.010	0.033–0.263	Supported
H3b	WT 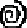 ESI	0.347^∗∗^	0.001	0.256–0.428	Supported
H3c	WT 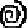 ESI ESB	0.098^∗∗^	0.001	0.053–0.154	Supported
H4	ESI 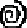 ESB	0.283^∗∗^	0.001	0.151–0.407	Supported

Note. ^∗^Significant at level *p* < 0.05 and ^∗∗^significant at level *p* < 0.01. Note. SN: subjective norms; ESA: E-shopping attitude; WT: website trust; ESI: E-shopping intention; ESB: E-shopping behaviour; CI: confidence interval.

## Data Availability

The data are associated in the manuscript.
